# Syria as a Residence for Consumptives

**Published:** 1860-05

**Authors:** 


*Syria as a Residence for Consumptives.*—The *Lancet* says, that Mr. Farley, late British Consul to Syria, points out the many advantages of Beyrout, as a residence for consumptive invalids. Its merits are, that while the climate is genial and the air exhilarating to a remarkable degree, the temperature is unvarying, and consequently the invalid can take out-door exercise almost every day in the year; besides this, the proximity of the Lebanon range enables the residents to avoid any inconvenience which may be experienced by delicate organizations, from the two hot months in the summer season. In that beautiful region every climate may be obtained, from that of England to that of Siberia, and the scenery is of the highest order of natural beauty. The cost of living there is slight, it is readily reached, and the society is agreeable.

